# Persistence is driven by a prefrontal motor circuit

**DOI:** 10.21203/rs.3.rs-2739144/v1

**Published:** 2023-04-20

**Authors:** Yihan Wang, Qian-Quan Sun

**Affiliations:** 1 Graduate Neuroscience Program, University of Wyoming, Laramie, WY82071, USA; 2 Department of Zoology and Physiology, University of Wyoming, Laramie, WY82071, USA; 3 Wyoming Sensory Biology Center of Biomedical Research Excellence, University of Wyoming, Laramie, WY82071, USA; 4 Lead contact

## Abstract

Persistence provides a long-lasting effect on actions, including avoiding predators and storing energy, and hence is crucial for the survival (Adolphs and Anderson, 2018). However, how the brain loads persistence on movements is unknown. Here, we demonstrate that being persistent is determined at the initial phase of movement, and this persistency will be sustained until the terminal signaling. The neural coding of persistent movement phases (initial or terminal) is independent from the judgement (i.e. valence) (Li et al., 2022; Wang et al., 2018) upon the external stimuli. Next, we identify a group of dorsal medial prefrontal cortex (dmPFC) motor cortex projecting (MP) neurons (Wang and Sun, 2021), which encodes the initial phase of a persistent movement rather than the valence. Inactivation of dmPFC MP neurons impairs the initiation of persistency and reduce the neural activity in the insular and motor cortex. Finally, a MP network-based computational model suggests that an intact, successive sensory stimulus acts as a triggering signal to direct the initiation of persistent movements. These findings reveal a neural mechanism that transforms the brain state from neutral to persistent during a movement.

## Introduction

Persistence is a fundamental trait that can help people achieving success in many areas, such as athletic and social competition (Ryans, 1938; Wolf, 1938). It provides a prolonged effect on the maintenance of motivation (Grant et al., 2007). It also contributes to the animal’s survival, such as the rank in social hierarchy and escaping from predators (Adolphs and Anderson, 2018). It can drive the actions successively from seconds to minutes and even outlasting the eliciting stimulus. Socially dominate animal exhibit effortful behaviors against their rivals (Zhou et al., 2017). Repetitive visual threat stimulus also gives a persistent state in *Drosophila* (Gibson et al., 2015). Although persistence has a great influence on both human and animal, there is a paucity of studies on its behavioral and neural mechanism. Here, we perform an investigation on how a persistent state is regulated in brain, and how it affects the movement output.

## Quantification of persistent movement

**Figure 1.**
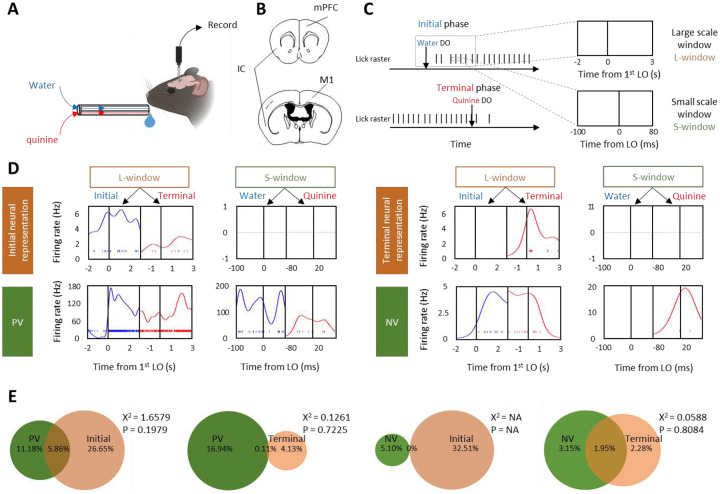
Dissociable neural representations of movement phases and valence A. Schematic of the behavioral setup. Water or quinine was continuously delivered through a multi-lick-ports with zero time delay on the delivery switches. Mice were trained to respond the liquid delivery without conditioning. B. Schematic of the recording sites. IC, insular cortex; mPFC, medial prefrontal cortex; M1, primary motor cortex. C. A representative task of persistent lick. Two recording windows were selected close to the water or quinine delivery onset (DO). For large scale window (L-window), the recording epoch started from 2s before the 1st water or quinine lick onset (LO) to 3s after the 1st water or quinine LO. For small scale window (S-window), the recording epoch started from 100ms before the LO to 80ms after the LO. D. Spike raster and firing rate estimation of four representative single-units, which were classified to represent initial phase and terminal phase of persistent movement, positive valence (PV) and negative valence (NV) at L-window and S-window, respectively. Spike raster and firing rate under water and quinine lick are colored in blue and red, respectively. E. Venn diagram of neural representations of valence and movement phases. The association of neural representations between the number of overlapping & non-overlapping fractions and the preference to these two features (valence or movement phases) was tested using Chi-square test. p > 0.05 indicates not significant association.

We first defined a persistent movement as continuously repeating of a single move (e.g. a cycle of tongue or limb moving) over 10 times. To impose a persistent movement on mice, water was deprived for 16 to 36 hours until the body weight decreased around 22% (Behavioral details, Methods). Mice were then head fixed on a customized set-up and trained to lick different types of liquid after the delivery (Fig 1A, Fig S1B, and Behavioral details, Methods), but received no other artificial sensory cues. Licking signals, facial and locomotor activities were measured. After training, we observed that mice displayed about 15–30s persistence on licking movement upon water delivery (Fig S1C–F). Consistent with the previously performed standard pattern of affective dynamics (Solomon, 1980), the lick frequency was maximized at the beginning phase (Fig S1C–F, right column, the peaks of lick frequency are indicated by black arrows) and then stabilized at 6 to 7 Hz until the end of delivery (Fig S1D–E) or the liquid switched to quinine (5mM, Fig S1C & F). Higher level of hedonic stimuli, 20% sucrose, won’t increase this frequency (Fig S1E–F). Furthermore, quinine delivery was more capable of terminating the persistent licking movement than the ending of water or sucrose delivery (p<0.05 in termination bias, Fig S1I). We thus used quinine to stop the licking movement.

We next quantified the examination window of valence and movement phases. For the valence, evaluating whether water or quinine is hedonic or aversive should base on the each contact (about 180ms around lick onset (LO), Fig S1C–F) to them. For the movement phases, an initiation or termination should happen between the stimuli and the behavior in a larger scale time window (about 5s from water or quinine DO to continuously licking starts or ends, Fig S1C–F). Therefore, we evaluated the neural firings of valence and movement phases at small (S, LO-100ms to LO+80ms) and large (L, 1^st^ LO-2s to 1^st^ LO+3s) scale window, respectively (Fig 1C).

## Dissociable neural coding of movement phases and valence

To characterize the single-unit that statistically represent valence and movement phases, we collected neural activity data from three brain regions, insular cortex (IC), which is known to encode valence in the taste system (Peng et al., 2015; Wang et al., 2018), primary motor cortex (M1), which represents motor commands of voluntary movement (Levy et al., 2020), and medial prefrontal cortex (mPFC), which has been shown to link those two brain regions (Wang and Sun, 2021). The single-units that were generated from neural activity data were then classified into different group of neural representations (Methods) based on how much they can discriminate liquid types (for valence) or movement phases.

We then asked whether the neural coding of movement phases and valence are associable or not by examining the fraction of neural representations of valence and movement phases in three brain regions (IC, M1, and mPFC). We found that 81.97% of neural representations of initial phase (≥65% in initial phase correlation) and 51.41% of neural representations of terminal phase (≥65% in terminal phase correlation) showed weak taste tuning (Fig 1D–E). 65.01% of neural representations of positive valence (PV, z>1.64 in water-licks correlation) and 61.76% of neural representations of negative valence (NV, z>1.64 in quinine-licks correlation) exhibited poor specification on movement phases (Fig 1D–E). By contrast, only small fraction (15.51%) of them displayed the preference to both valence and movement phases (Fig 1E), though this number trivially varied from regions to regions (Fig S4).

To test the possibility that the neural networks of representing movement phases and valence may interact with each other, we examined the connectivity between non-overlapping initial phase and PV neural representations using total spiking probability edges (TSPE) (De Blasi et al., 2019) and compared it with shuffled connectivity. Our results showed that no overall excitatory impact from initial phase to PV or from PV to initial phase neural representations (mean of real TSPE < 99 percentile of shuffled TSPE, Fig S5E). Note that the connectivity between terminal phase and NV neural representations was not available because their spiking data were not able to construct cross-correlation in 50ms-time window, suggesting the coding of terminal phase and NV are not associated. Together, these results suggest that the movement phases and valence are encoded separately.

## Coding of initial phase in dmPFC MP neurons

**Figure 2.**
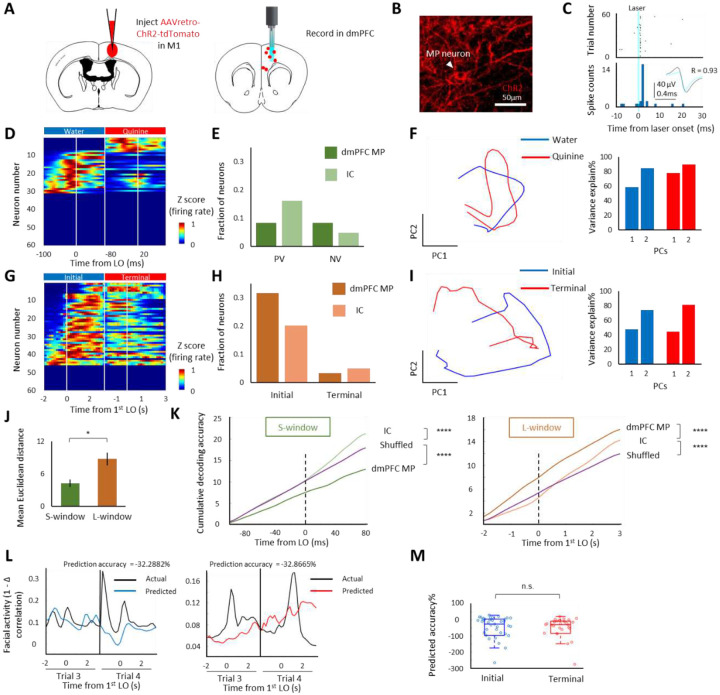
Coding of initial phase in dmPFC MP neurons A. Schematics showing the labeling and recording of MP neuron in dmPFC. B. Representative image showing channelrhodopsin-2 (ChR2) expression in MP neuron. C. Identification of labeled MP neuron. We identified the unit as MP neuron when there was a significant probability of evoked spikes, appeared from 0 to 5ms after laser onset (light blue), and when there was a high correlation (R>0.85) between evoked spikes (light blue waveform) and other spikes (black waveform). D & G. Color-coded plot showing MP neural response in S-window (D) and in L-window (G) from one representative trial. E & H. Fraction of valence (E) and movement phases (H) classified neural representations in MP and IC neurons. F & I. Left, PCA trajectories of dmPFC MP neuron. Right, bar plot showing cumulative variance explained percentage of first two PCs. J. Comparison of mean Euclidean distance, from PCA trajectories of dmPFC MP neuron, between S-window and L-window. K. Decoding of taste signals (water or quinine) in S-window (left) phase (initial or terminal) in L-window (right). L. Examples of facial activities (black traces) overlaid with MP firing predictions (Left, initial phase; right, terminal phase). M. Summary of facial activity predictions by recorded MP neurons. *P < 0.05, ****P < 0.0001, n.s. p > 0.05

Next we investigated the neural basis that represented the initial phase of persistent movement. We first compared the fraction of clustered neural representations in the IC, M1, and mPFC. Second, we examined the decoding performance in these brain regions by training a linear discriminant decoder on firing rate data. We found that dmPFC MP neurons (Wang and Sun, 2021) exhibited the best representation of initial phase among the three mapped brain regions (Fig 2, Fig S4, S6, and S7). Specifically, the results of neural clusters mapping showed that 32% of dmPFC MP neurons exhibited an extent (≥65% shuffled activity at initial phase & ≤35% shuffled activity at terminal phase) of representation of initial phase (Fig 2H), while only 3% of them showed a degree (≥65% shuffled activity at terminal phase & ≤35% shuffled activity at initial phase) of representation of terminal phase (Fig 2H). No more than 20% of dmPFC MP neurons showed a degree (z>1.64 in valence correlations compared to shuffled activity) of valence representation (PV+NV, Fig 2E). The results of decoding performance showed that dmPFC MP neurons displayed poor capability to discriminate positive and negative valence (p<0.0001 lower than shuffled cumulative decoding accuracy, Fig 2K **left**), while exhibited high representation of movement phases (p<0.0001 higher than IC and shuffled cumulative decoding accuracy, Fig 2k **right**). To confirm the discriminability of dmPFC MP neuron between valence and movement phases, the separations of positive and negative value at S- and L- window were compared. We first embedded neural population activity at S- and L-window of dmPFC MP neurons into trajectories by principal component analysis (PCA) and then measured Euclidean distances between the trajectories. The results showed that the separation of PCA trajectories at L-window was significant higher than it at S-window (p<0.05, Fig 2J).

In rodents, facial activity can reflect bodily arousal (Dolensek et al., 2020). In the persistent licking task, the facial activity showed a reliable increase right after water delivery onset (DO) (p<0.05 compared to the baseline in all sessions, Fig S1K–N). We, thus, used facial activity to assess bodily arousal at the initial phase. To examine whether the firing changes of dmPFC MP neuron at initial phase is due to bodily arousal, we trained a Hammerstein-Wiener model and tested its prediction accuracy on facial activity data. Although some mPFC neurons exhibited a degree of facial activity representation at initial phase (p<0.05 initial phase (median=−4.5671; 21/194 neurons with accuracy>30%) vs terminal phase (median=−36.9341; no neuron with accuracy>30%), Fig S8D2), all dmPFC MP neurons showed poor prediction performance at both initial (median=−14.5639; no neuron with accuracy>30%) and terminal phase (median=−27.4588; no neuron with accuracy>30%) on facial activity and no significant difference between these two phases (p>0.05, Fig 1M).

## Effect of dmPFC MP neuron silencing on persistent movement

**Figure 3.**
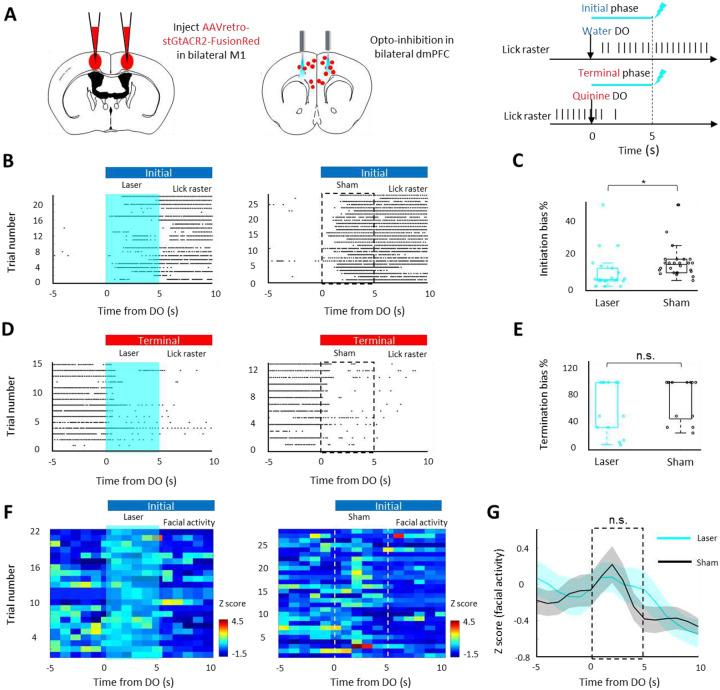
dmPFC MP neuron is required for the initiation of persistent licking movement **A**. Left: schematic of bilateral silencing MP neurons in dmPFC. Right: experimental design for optogenetic silencing. Mice received laser or sham stimulation (5s, 20Hz) concurrent with water or quinine DO. **B & D**. Raster plot showing licking movement relative to water DO (B) or quinine DO (E). Laser (left) or sham (right) was triggered by DO. **C & E**. Percentage of bias that started (D) or stopped (G) persistent lick (see Methods). **F**. Color-coded plot showing facial activity relative to water DO. **G**. Z scored facial activity relative to water DO. Values are mean ± s.e.m. *p<0.05, n.s. p>0.05.

To test the role of dmPFC MP neuron on persistent licking movement in three phases (initial, middle, and terminal), we examined the initiation and termination bias, as well as lick frequency. Our results showed that optogenetic silencing of dmPFC MP neurons impaired licking initiation (p<0.001 compared to the sham group, Fig 3C) but did not affect the termination of lick (p>0.05 compared to the sham group, Fig 3E) or the lick frequency at the middle phase (Fig S10B2) in thirsty mice. The similarity of the thirsty level was confirmed by comparing the body weight decrease within sham and laser group (**Fig SB4**, **C3**, and **D3**). To further confirm the function of dmPFC MP neuron on licking initiation, dmPFC MP neurons were chemogenetically silenced and the licking chances were tested. As expected, thirsty mice with chemogenetically silenced dmPFC MP neuron had lower chance to drink water (p<0.05 compared to the saline group, Fig S9C). To examine whether the dampened licking initiation was due to a decrease in overall bodily arousal, we tested the facial and locomotor activity with or without optogenetic silencing. Mice in the sham and laser group displayed the similar facial and locomotor activity level at all initial, middle, and terminal phase (Fig 3G and Fig S10B–D), suggesting that the dampened licking initiation were not due to the reduction of bodily arousal. Next, we asked whether the effect of dmPFC MP neuron silencing is specific on the licking initiation or also general on other type of behavioral initiation. Mice showed a period of persistent running after a mild electric shock (Fig S9H). To test if inactivation of dmPFC MP neurons also impairs this behavior, we examined the body activity after giving a 1s electric tail shock. Indeed, optogenetic silencing of dmPFC MP neurons suppressed the escaping behavior (reduced facial and locomotor activity at the initial phase of escaping, Fig S9H–I), which suggests that dmPFC MP neuron involves in the initiation of persistent movements in general. This result is in agreement with previously reported overall dmPFC neurons, which were showed to delay the initiation of avoidance (Jercog et al., 2021).

**Figure 4.**
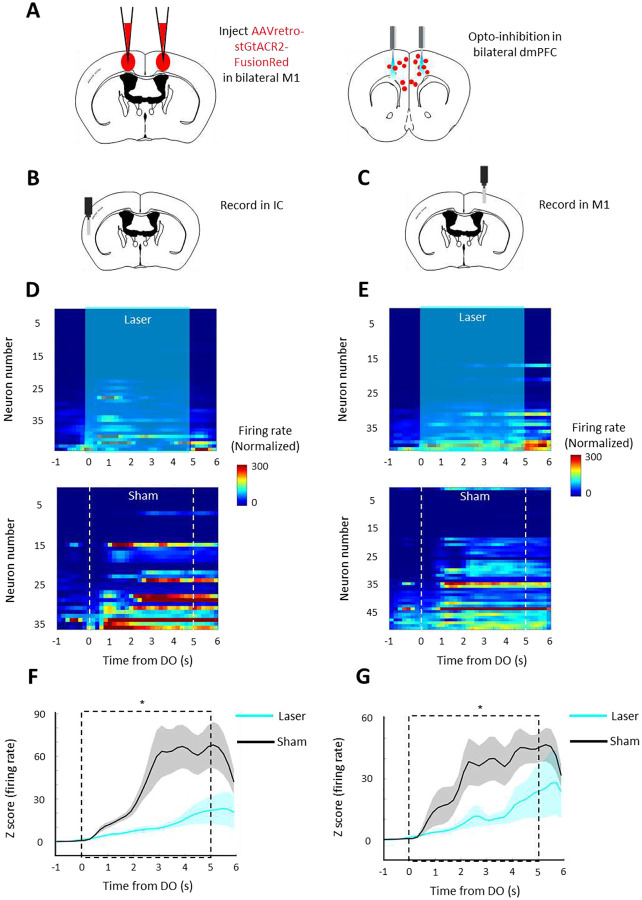
Silencing of dmPFC MP neuron impairs the neural activity in motor and insular cortex **A**. Schematic of bilateral silencing MP neurons in dmPFC. **B-C**. Schematic of recording sites in IC (B) and M1 (C). **D-E**. Baseline subtracted, z scored firing rate, relative to water DO, for the recorded neurons in IC (D) and in M1 (E) from one representative trial. Laser (left) or sham (right) was triggered by DO. **F-G**. Mean baseline subtracted, z scored firing rate, relative to water DO, of IC (F) and M1 (G) neurons. Values are mean ± s.e.m. *p<0.05.

We next hypothesized that if silencing of dmPFC MP neuron impairs the persistent licking initiation, then the brain state of positive valence and tongue moving should decrease accordingly. IC is thought to encode taste valence (38/42 in w-q vs 11/42 in w-s vs 33/42 in w-w of mean decoding accuracy higher than chance, Fig S6A) (Peng et al., 2015; Wang et al., 2018), while M1 was involved in voluntary control of tongue movement during the lick (12% neurons with z>1.64 in lick correlations compared to shuffled activity, Fig S4B1)(Martin et al., 1997; Yao et al., 2002). Therefore activities in IC and M1 should be affected by the optogenetic silencing of dmPFC MP neurons. To test the hypothesis, we measured the neural activity in these two brain regions by shined with or without laser on dmPFC. As we expected, M1 and IC neural activity were all decreased (p<0.05) after dmPFC MP neurons were optogenetically silenced (Fig 4F & G). We further confirmed this initial phase specificity by excluding the effect of silencing of dmPFC MP neuron on valence at middle phase (no significant difference of sucrose-lick frequency between sham and laser group, Fig S10A). Collectively, our results suggest that dmPFC MP neuron is required to start continuous licks, and further constructing taste valence during the initial phase, but lose its necessity upon the persistent movement already initiated.

## A MP network-based computational model

**Figure 5.**
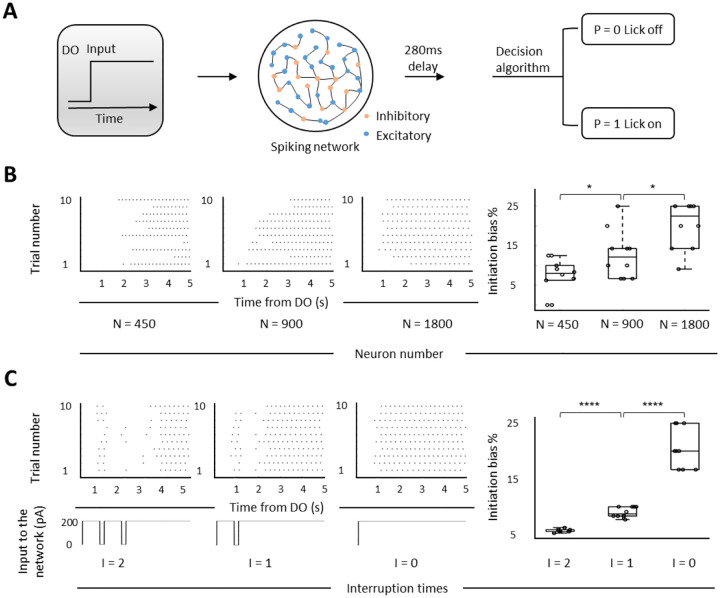
Modulation of neuron number and input in the MP network-based model **A**. The MP network-based model. A spiking network receives an input of current after the DO with a flexible (1 to 200ms, for panel B) or fixed (50ms, for panel C) delay. 56% connectivity is excitatory and 44% of it is inhibitory. The lick raster outputs are calculated by a decision algorithm (see Methods) using the simulated spike data. **B**. Simulation of optogenetic inhibition of dmPFC MP neurons by reducing the neuron number in the MP network based model. Left 1–3: lick raster produced by the model under indicated neuron number. Right: percentage of initiation bias that calculated from left lick raster data. **C**. Performance of licking behavior under the different continuities of input current. Left 1–3: top: lick raster plots under, bottom: inputs with indicated interruption times. Right: percentages of initiation bias with indicated interruption times. *p < 0.05, ****p < 0.0001

Finally, we asked what triggers the MP network to execute a persistent movement. To answer this question, we built a neural network-based model (Fig 5A) and examined how the output of licking performance changes in response to different types of inputs. The design of this model was mainly based on two criteria as following: (1) the inter-spike-interval of a single neuron in the model are adapted to the MP neuron in mPFC (Fig S11A); (2) the neural population of modeled network performs a rotational dynamic (Linden et al., 2022), as we assumed that the tongue move is in a rhythmic pattern (Fig S11B–C). To verify if the output of this model is in agreement with the performance of thirsty mice in actual experiment, we manipulated the firing rate of simulated network by inserting the inputs with different amplitudes and examined the output of lick frequency and initiation bias. We found no linear relationship between the mean neural firing rate and above two metrics (Fig S11D, red) resembling experimental observations (Fig S11D, black). To further ensure the viability of the MP network-based model, we simulated the optogenetic silencing of dmPFC MP neurons through decreasing the number of neurons in the modeled network. Consistent with the experimental data, the neural population reduced network inhibited the initiation of persistent lick (Fig 5B). We then evaluated the temporal continuity effect of the input on the network output. We found that even a single short-term (200ms) interruption disrupted the initiation of continuous lick (average 57% decrease on initiation bias, Fig 5C). The bias percentage of twice interruption plummeted to the 5.7±0.08% (vs 21±1.17% of no interruption, Fig 5C). This temporally continuous input of the model suggests that the triggering signal to MP network should be an intact, continuous sensory stimulation.

## Summary and Discussion

Our study identified a neural circuit, which is responsible for directing the initiation of persistent movements. Upon receiving a sensory signal, dmPFC MP neurons can send the command signals to the primary motor cortex, which in turn initiates downstream machineries for a persistent movement. Our findings suggest that the decision to proceed to a persistent movement is not relied on an acute judgement of the present situation.

### Summary of neural principles.

Based on the membrane properties and network connectivity of MP neuron, we summarized two neural principles that directs the initiation of persistent movement. First, the circuits should be composed of slow spiking neurons. mPFC layer V MP neurons are the main functional units linking deep brain regions and motor cortex, and they fire slower than layer II pyramidal neurons (van Aerde and Feldmeyer, 2015; Wang and Sun, 2021). In line with the observation of maximum lick frequency (about 8 Hz) during initial phases (FigS1C–F), the firing rate of the tongue modulating neurons should not surpass this level. Second, the circuit should be constructed with monotonous outputs and diverged inputs. Within the mPFC, the projection of MP network is relatively sparse, as it only project to contralateral dmPFC and motor regions, while the inputs to the MP network come from various deep brain regions (Wang and Sun, 2021). This connection configuration may help to not only simultaneously receive the signals from the neurons that tune multiple aspects of sensory cues, but also regulate downstream motor output independent of other neural networks.

### Formation of generalized command.

Although we revealed a neural basis encoding the initial phase of persistent movements, it is unclear how the generalized command forms or how specific movement execute under a persistent pattern: first, how to recognize the valued aspects of sensory cues? This unsupervised learning process requires a generalization neural network (Poggio and Bizzi, 2004) extracts only few optimal aspects of stimulus while keeping the specificity at the same time. The question is why some of the aspects are optimal but not the others. We speculate that these optimized aspects are the ones most resembling or temporal closest sensory cues to the reward or punishment, as they can provide an efficient and direct link to a certain behavioral pattern. Second, how to associate the sensory cue with certain movement pattern? Based on the reciprocal connection between mPFC and motor cortex (Wang and Sun, 2021), we reason that a feedback loop between the generalization network and the motor regions may be required. Meanwhile, a valence input may be also necessary to construct the association and sensitize the response to sensory cue.

### Explanation of the dynamics of valence coding.

To explain the trial bias (Fig S4A1 & B1 & C1) and gradually faded firings (Fig S4D–E) of PV neural representations during water licks, we postulate two possibilities: (1) Transformation of neural representations. Since some neural representations play as dual role at initial phase, decreasing firing rate and transform to other representations at the following phases may reduce the effect of generalization. (2) Valence decreases. Since the thirsty mice may become sated along with the lick proceeding, the value of water will decrease accordingly to the mice.

## METHODS

### Subject details

All experimental procedures were approved by the Institutional Animal Care and Use Committee (IACUC) and the Biosafety Committee of the University of Wyoming. 20 male and female immune-competent mice at specific ages (indicated in **Surgeries** and **Behavioral details**) were used for different experimental purposes. All of them were bred on a C57/BL6J background. Mice older than 30 days were housed with same sex littermate or housed alone in a vivarium maintained at 21–23 °C on a 12-hour light/dark cycle. Mice after the electrode or head bar implantation were housed alone. For chemo-genetic experiments, 4 mice received AAV injection and head bar implant. For optogenetic experiments, 8 mice received AAV injection, opto-electrode, and head bar implantation. 4 mice were implanted with electrodes and head bar. 2 mice were implant with optic fibers and electrodes. 1 mouse were implant with optic fibers. For all behavioral experiments, mice were deprived of water before the experimental day for 16–36 hours until 75–80% of their free-feeding body weight. After each behavioral experiment, mice were back to their home cage with unlimited access to water for at least five days until the next behavioral test day.

### Surgeries

The preparatory procedures are similar for both implantation and injection. Mice were anesthetized using oxygenated (2 LPM for induction while 0.4 LPM for maintenance) 2% isoflurane (v/v). Mice were head fixed with a stereotaxic device (NARISHIGE SG-4N) and maintained at 37 °C with a heat pad (K&H no. 1060). Seventy percent isopropyl alcohol and iodine were placed on the incision site. The skull was exposed by cutting the skin and removing the dura and connective tissue. The coordinates, used for positioning injection and implant sites, were relative to Bregma (Antero-posterior A-P, Medio-lateral M-L, Dorsal-ventral D-V) in mm. After surgeries, mice were administrated intraperitoneally with ibuprofen (50mg/Kg) and maintained at 37 °C for 30–60 mins before returning to the home cage.

For viral injection, P14–30 mice were used. A small craniotomy (around 0.2mm diameter) was made over the injection site. The delivery glass filament (Drummond Scientific Co.) with around 5 μm tip diameters were backfilled with 2 μL viral solution. Pressure injection using a customized device driven by single axis hydraulic manipulator (NARISHIGE mmo-220A) delivered viral solution (undiluted, 100 nL at each injection site) into the desired regions at a rate of 30–50 nL/min. Opto-tagged mPFC MP neurons were labeled using pAAV-CAG-hChR2-mcherry (Addgene_28017-AAVrg) injected into motor cortex (A-P −0.6, M-L 1.0, D-V 0.2 0.5 0.8). For chemogenetic inhibition, pAAV-Ef1a-mCherry-IRES-Cre (Addgene_55632-AAVrg) were injected into bilateral motor cortex (A-P −0.6, M-L ±1.0, D-V 0.2 0.5 0.8), followed by injecting pAAV-hsyn-DIO-hm4D(Gi) (Addgene_44362-AAV5) into bilateral dmPFC (A-P 1.35, M-L ±0.2, D-V 0.2 0.5 0.8 at an angle of 30° from upright). For optogenetic inhibition, pAAV-CKIIa-stGtACR2-FusionRed (Addgene_105669-AAVrg) was injected into bilateral motor cortex (A-P −0.6, M-L ±1.0, D-V 0.2 0.5 0.8). Mice were returned to the home cage until at least three weeks before they were used for the implantation.

For implantation, silicon probes (A4×8-Edge-2mm-100-200-177-CM32 or A1×32-Edge-5mm-25–177-CM32, NeuroNexus) or 32-tetrode bundles (Bio-Signal technologies) were implanted followed by optic fibers and head bar. To build opto-electrode, optic fibers (MFC_200/245–0.37_2.0mm_MF1.25_FLT) were fixed around 0.5 mm above the electrodes using crazy glue and dental cement (Lang Dental). In opto-inhibition experiments, two optic fibers (MFC_600/710–0.37_1.0mm_MF1.25_FLT) were implanted to the bilateral prefrontal cortices (A-P 1.7, M-L ±0.5, D-V 0.5). To fit the shape of prefrontal, motor, and insular cortex, customized 32-tetrode bundles were split into one or two clusters. To record the single unit, electrodes were implanted in the left hemisphere with the following designs and coordinates: silicon probe (A1×32-Edge −5mm-25–177-CM32) was implanted at the pIC (A-P −0.5-(−1.5), M-L 3–4, D-V 3); silicon probe (A1×32–5mm-25–177-CM32) was implanted at the aIC (A-P 1.5–1.7, M-L 2.5–3.5, D-V 2.5); silicon probe (A4×8-Edge-2mm-100-200-177-CM32) based opto-electrode was implanted at the mPFC (A-P 1.0–1.7, M-L 0.1–1.5, D-V 1); 32-tetrode bundles (one-cluster) based opto-electrode was implanted at the mPFC (A-P 1.0–1.7, M-L 0.1–1.5, D-V 1); 32-tetrode bundles (two-cluster) were implanted at the pIC (A-P −0.5-(−1.5), M-L 3–4, D-V 3) and motor cortex (A-P 1–2, M-L 1–2, D-V 0.5). During the surgery, the skull was horizontally aligned through a fixing apparatus (Stoelting Co.). An anchor screw was placed on the right cerebellum to connect ground wires of the electrodes. After placing the anchor screw and electrodes, silicone sealant (kwik-cast, world precision instrument) was applied above the exposed brain tissue. A customized head bar (github.com/ywang2822/Multi_Lick_ports_behavioral_setup) was then positioned over the skull. To affix the implant, Metabond (C&B Metabond, Parkell) and dental cement (Lang Dental) were applied. The behavioral experiments started at least one week after the surgery.

### Behavioral details

The head-fix setup was connected to a construction rod (Throlabs) by a 3d printed connector (github.com/ywang2822/Multi_Lick_ports_behavioral_setup). Multi-lick-ports (github.com/ywang2822/Multi_Lick_ports_behavioral_setup) were placed in front of the head fix and connected to the Dual Lick Port Detector (www.janelia.org/open-science/dual-lick-port-detector). Three Clearlink sets (Baxter) were used for liquid delivery. The delivery speed was manually calibrated to 0.15 – 0.2mL/min every time before the behavioral test. The delivery switch was controlled by three solenoid valves (LFVA1220210H, THE LEE CO.) in a noise-reducing box. The switch timing was programmed through the Bpod (Sanworks). The signal of mice locomotor activity was collected through an optical shaft encoder (H5–360-IE-S, US digital). For facial videography set up, the camera (S3-U3-91S6C-C, Teledyne FLIR) was positioned at the right side of the mouse’s lateral face surface, which illuminated by two infrared arrays. For laser delivery, a solid-state laser (Shanghai Laser& Optics Century Co., 473 nm) was connected to fiber optic patch cord (Doric Lenses), which attached to the implanted optic fibers using ceramic mating sleeves. To conditionally control the laser delivery by water, sucrose, or quinine onset, we used a 4-way data switch box (BNC, Kentek) to bridge the laser and solenoid valves. A programmable stimulator (A-M system, model 4100) was used to control laser delivery and a voltage pulse for tail shock experiment. All signals, including frame timing, wheel speed, liquid delivery timing, lick timing, shock timing, and laser delivery timing, were sent to an USB interface board (Intan Technologies, RHD).

For licking task, mice were deprived of water at least seven days after the surgery. The deprivation was terminated until the body weight decreased approximately 22%. During training phase, mice learned to sense the water through their whiskers. We considered mice to become proficient at the task when licking happened within 3s after the delivery onset (DO) in all repeated trials. During the test phase, we first delivered water and 20% sucrose in a random sequence for a total of 30s. After at least 5 min, we then orderly delivered water, 20% sucrose, and 5 mM quinine for 10s each or water and 5 mM quinine for 15s each.

For tail shocking task, 16–23 volts electrical shocks were administered to the tail by a customized shocker (electric shock box machine kit, STEREN). Two conductive adhesive copper tapes were connected to the shocker and positioned 2 cm apart at the tail by sticking on customized heat shrink tube (various on the circumference of mouse tail). During the first time of training, the voltage of electrical shocks were adjusted until escaping behavior was observed (speed>10 cm/s right after the shock). This voltage was recorded and used for the following tests. Those who did not perform escaping behavior were excluded from the test.

### Optogenetic inhibition

We illuminated bilateral prefrontal cortices using 473 nm laser to activate stGtACR2. Laser pulses (40ms width at 20Hz) were delivered in a 5s duration. The onset of laser pulses was triggered based on either water DO or quinine DO. The optogenetic inhibition experiments were only performed after mice reached stable behavioral level (after at least two test phases and (lick onset (LO) – DO < 3s) in all test phases). The trials, of which lick frequency > 0.5 Hz in the time course 3s before DO, were excluded. Histological characterizations were used to identify the viral infection.

### Optogenetic identification

We applied laser pulses (1ms width at 20 Hz, 3s duration) on the unilaterally prefrontal cortex of viral (AAV-ChR2) injected mice. Laser and network-evoked spikes (see also Spike sorting and firing rate estimation) were identified using the Stimulus Associated spike Latency Test (SALT, (Kvitsiani et al., 2013)). Specifically, laser and network-evoked spikes were assessed in a 0–5ms and a 6–10ms temporal window after laser onset, respectively. For those units with significant correlation (correlation coefficient > 0.85) of average waveform and significantly different distribution (P < 0.05) of spike latency with baseline units were identified as laser or network-evoked units.

### Chemogenetic inhibition

Viral pAAV-hsyn-DIO-hm4D(Gi) (Addgene_44362-AAV5) injected mice were administered intraperitoneally with Clozapine N-oxide dihydrochloride (CNO, 2mg/kg, Tocris) ten minutes before the licking or tail shocking task. Only the mice reached stable behavioral level (after at least two test phases and (LO – DO < 3s) in all test phases) were used for chemogenetic experiments. In the licking task, mice were re-trained to lick the water one to two times after recovery from CNO administration. The re-trained phases were not included in test phases.

### Analysis of facial and locomotor activity

We collected the frames during the licking or tail shocking task. We then converted these frames into histogram of oriented gradients (HOG) vectors by using 8 orientations, 32 pixels per cell and 1 cell per block. To extract the most variant facial part, we cropped the ear part with 364×296 pixels fixed size and manually selected position of each transformed HOG vector (Dolensek et al., 2020). Temporally adjacent HOG vectors were paired, the facial activity at each time point was calculated as follows: 1-ΔR, where ΔR is the correlation coefficient between two temporally adjacent HOG vectors.

The signals that collected from the encoder were digital pulses. The locomotor activity was calculated as speed $(\text{cm}/\text{s}):\frac{\text{circumf}}{CPR\cdot dt}$, where *circumf* is the circumference (cm) of the wheel, *CPR* (cycles per revolution) is 360, and *dt* is the time interval between two digital pulses.

### Analysis of licking initiation/termination bias

With the feeling of extremely thirsty, the mice will start a non-stop licking behavior when water is available until feeling satiated or the delivery stopped (Allen et al., 2019) (Fig S1D). To evaluate if the mice start or stop the continuous, but not discrete, lick, we calculated the initiation and termination bias. We first calculated 25 simple moving averages (SMA) on the time duration of 5s after DO as following: $SMA=\sum_{i=1}^{n}\frac{l\times dt}{n}$, where *l* is the lick times during the time duration *dt*, which is set as 200ms, and n, which is set as 5, is the number of *dt*. For the initiation bias, all values of *SMA* were ignored if there was a zero value after DO. The SMA value was counted from the last non-zero value. The initiation bias (ibias) was calculated as: $\text{ibias}=\frac{1}{idx}$, where *idx* is the first time point of the SMA > 1.2 (6Hz). If all SMA values equal zeros, *ibias* was set as zero. For the termination bias, the SMA value was counted from the first time point after the quinine DO (for the water-quinine and water-sucrose-quinine session) or the end time point of water delivery (for the session water-water session) or sucrose delivery (for the session water-sucrose session). The termination bias (tbias) was calculated as: $\text{tbias}=\frac{1}{idx}$, where *idx* is the first time point of the SMA < 1 (5Hz). If all SMA values ≥ 1, *tbias* was set as zero.

### Spike sorting and firing rate estimation

Before spike sorting, single unit data were acquired from 32-channel RHD head stage, which connected with a signal acquisition system (USB board, Intan Technologies) with sampling rate at 20 kHz. All spike sorting procedures were performed with an offline software Spikesorter (Swindale and Spacek, 2014, 2015, 2016) (swindale.ecc.ubc.ca/home-page/software/) under following parameters: (noise calculation: median; threshold: 80μV, 5x noise, 0.75ms window width) for even detection and under following parameters: (pca dimensions = 2; template window: −0.8 to 0.8; starting sigma = 5; threshold = 9) for clustering. We used Bayesian adaptive kernel smoother (Ahmadi et al., 2018) with following parameters, α = 4 and β = (number of spike events) ^ (4/5), to estimate the firing rate of sorted spikes. For small scale temporal window (180ms), we used a bandwidth of 5ms. While for large scale temporal window (5s), we used a bandwidth of 200ms.

### Cell classification

For the cell classification to discriminate water- and quinine-licks, we categorized single-units into four separate groups of neural representation (lick, positive valence (PV), negative valence (NV), and mixed valence (MV)) based on firing rate estimation at the lick window (LO-100ms: LO+80ms). To determine if the firing rate is significantly higher than normal condition, we created pseudo-trials that have the same lick interval with the corresponding real licks during the 10s baseline. Individual time bins of each pseudo lick trials were concatenated horizontally and shuffled. This procedure was repeated 1000 times and the pseudo-trial matrix was calculated as the mean among shuffled datasets. For real lick trials, we only selected the first four trials for encoding analysis. At each individual time bin of pseudo and real trials, we calculated Euclidean norm of two temporally adjacent firing rate estimations (5ms each). When absolute z-scores of two distributions ($Z_{12}=\frac{\mu_{1}-\mu_{2}}{\sqrt{\sigma_{1}^{2}+\sigma_{2}^{2}}}$, where *μ* and *σ* represent mean and standard deviation, respectively, of the distribution1 and 2) exceeded 1.29, they were considered significantly different. We selected single-units with significantly high firing rate in water or quinine lick trials for further analysis. To evaluate the time bias of firing rate across lick trials, we mean centered the whole firing rate matrix. The Frobenius norms were calculated as follows: $\text{norm}=\sqrt{\sum_{i=1}^{n}{\left(t_{i}-c\right)}^{2}}$, where *t*_*i*_ is the mean column value of firing rate matrix with 10ms time bin across lick trials, *c* is centered mean, and n equals the number of time bins. We categorized a single-unit with the time bias of lick trials when its real norm greater than 95% of 1000 shuffled norms. To estimate if the peak of firing rate of two distributions is different, we compared the times of the maximum firing rate across lick trials between two distributions using two sample t-test. Their firing rate peaks were considered different when p-value less than 0.05 (z-score>1.64). Single-units were categorized into the group of lick, positive or negative valence, or mixed response when their firing rates met following conditions: lick, quinine trials > pseudo trials & water trials > pseudo trials & without time bias of the firing peak between quinine trials and water trials & with the time bias of water and quinine trials; positive valence (PV), water trials > pseudo trials & water trials > quinine trials & quinine trials ≤ pseudo trials; negative valence (NV), quinine trials > pseudo trials & quinine trials > water trials & water trials ≤ pseudo trials; mixed valence (MV), water trials > pseudo trials & quinine trials > pseudo trials & with time bias of the firing peak between quinine trials and water trials & with the time bias of water and quinine trials; others were grouped into unrelated valence (UV).

To examine if there is a trial bias of firing rate during the water-licks, we compared the spike times at the small scale time window of water-licks. The matrices were binned (8 trials per group) and the mean values of each group were calculated. New generated binned firing rate matrices were then used. We next calculated Frobenius norm: $\text{norm}=\sqrt{\sum_{i=1}^{n}{\left(t_{i}-c\right)}^{2}}$, where *t*_*i*_ is the mean row value of the binned firing rate matrix, *c* is centered mean, while *n* is the number of trials. When the real Frobenius norm was greater than 95% of 1000 shuffled norm, the single-unit was considered with trial bias in water-lick trials.

For the cell classification to discriminate initial and terminal phase of persistent movement, we assessed the firing rates at the 2s temporal window before or after the first water or quinine LO. The Five time point (0s, 0.5s, 1s, 1.5s, and 2s) were used for classification analysis. To construct pseudo data, the over 70s spike train data were used to extract five-time point random temporal window. We defined a single-unit with estimated firing rate higher than 65% pseudo data at the temporal window (1^st^ water LO-2s: 1^st^ water LO) and lower than 35% pseudo data at the temporal window (1^st^ quinine LO: 1^st^ quinine LO+2s) as initial phase neural representation before LO; with estimated firing rate higher than 65% pseudo data at the temporal window (1^st^ water LO: 1^st^ water LO+2s) and lower than 35% pseudo data at the temporal window (1^st^ quinine LO: 1^st^ quinine LO+2s) as initial phase neural representation after LO; with estimated firing rate higher than 65% pseudo data at the temporal window (1^st^ quinine LO: 1^st^ quinine LO+2s) and lower than 35% pseudo data at the temporal window (1^st^ water LO-2s: 1^st^ water LO+2s) as terminal phase neural representation. Others were classified as neural representations of unrelated movement phases.

### Connectivity estimation

To estimate the connections among different neural representations, we used Total Spiking Probability Edges (TSPE (De Blasi et al., 2019)). This method allows us to calculate the cross-correlation between pairs of spike trains and to evaluate excitatory connections. Furthermore, it gives high accuracy estimation in a short recording period. However, this method can only be used for the comparison of two individual single-units. To apply this method on two networks, we first selected spike trains of photo-tagged and network evoked single-units. We assumed the connection from photo-tagged to network evoked single-units is positive. For the individual single-unit, 3s time duration of spike times data was cropped and sent to TSPE calculation. We next selected spike trains of the neural representations of NV, PV, and initial phase in three brain regions (IC, M1, and mPFC). Individual time windows of 180ms across the lick trials (LO-100ms: LO+80ms) were extracted and recombined. The connectivity among these neural representations was calculated using TSPE. To perform a statistical test, we compared the real TSPE and the pseudo TSPE, in which 3s spike times data were shuffled 200 times and created pseudo spike times. 70, 80, 90, 95, and 99 percentile of pseudo TSPE were used for the comparison based on the neural representation number of real data. Specifically, if the real data number is less than 10, they will not be used for the comparison; if the real data number is between 10 and 15, 80 percentile of pseudo TSPE will be used for the comparison; if the real data number is between 15 and 20, 90 percentile of pseudo TSPE will be used for the comparison; if the real data number is between 20 and 32, 95 percentile of pseudo TSPE will be used for the comparison; if the real data number is larger than 32, 99 percentile of pseudo TSPE will be used for the comparison.

### Decoding analysis

For both small and large scale temporal window decoding, we employed a multiclass linear discriminant analysis. We first estimated spike firing rate (see Spike sorting and firing rate estimation) in water and quinine lick window (LO-100ms to LO+80ms for small scale window and first LO-2s to first LO+3s for large scale window). The firing rate was normalized by dividing the maximum firing rate value of water and quinine lick window. To create firing rate pseudo data, pseudo LOs were randomly selected for the entire time duration of the spike train. This procedure was repeated 50 times. Same with real data normalization, the pseudo data were divided by the max firing rate value in each repeat. To improve the subsequent decoding performance, we reduced the dimensionality of the neural activity by PCA. The dimensions that explained over 85% of the data variance were selected to train a decoder, which is based on an error-correcting output codes (ECOC) classifier using binary support vector machine (SVM) learner (MATLAB ‘fitcecoc’ function). 50 percent of real and pseudo data were used to train the decoder, and the rest of them were used to test the decoder’s performance. This procedure was repeated 10 times to get an average accuracy and standard deviation.

### Facial activity prediction

We trained Hammerstein-Wiener model to predict behavioral activity using estimated firing rate (Ethier et al., 2012). Before the modeling, the firing rates were estimated in a large scale temporal window (first LO-2s to first LO+3s) and normalized into a range from 0 to 1 by dividing the maximum firing rate value of water and quinine lick window. The facial dynamics (see **Facial activity analysis**) were smoothed using Gaussian filter. The parameters that can best simulate facial dynamics were used to construct Hammerstein-Wiener model. Specifically, the number of zeros was set in a range from 0 to 2 (nb-1); the number of poles was set in a range from 1 to 3 (nf); and the degree of input nonlinearity estimator (one-dimensional polynomial) was set from 2 to 5. We then used MATLAB function ‘predict’ to obtain decoding accuracies of facial dynamics from the test spike data. Specifically, the data (four trials in total) were split 50/50. First two trials were used for training the model and last two trials were used for testing.

### Histology

To check the position of implanted electrodes and site of injection, mice were anesthetized with 2% isoflurane (v/v) and perfused intracardially with 0.9% saline followed by 4% paraformaldehyde (PFA). Fixed brains were washed three times before dehydration in 30% sucrose for 24 hr. Slices were cut on a cryostat (MICROM, HM505E) at 70μm thickness after embedding with an optimal cutting temperature compound (Tissue tek). Fluorescent images were acquired by an LSM 980 microscope (Zeiss), with a 10 × 0.45NA objective or a 2.5 × 0.085NA objective.

### MP network-based model

The purpose of this modeling was to create lick raster readouts through giving a type of current input, simulating a network of neural activities, and transforming the simulated spike timing to the lick timing.

For the input current, we set the starting time ***t***_***s***_ with a flexible (1–200ms) or a fixed (50ms) delay after the DO. The amplitudes of input current were varied from 50–200pA according to the different simulations. To test the effect of input continuity, the current was cut using one or twice 200ms zero amplitude.

We simulated neural activity using Brian2 simulator package in a customized python code based on a sparsely connected spiking neuron network (Brunel, 2000). The network consisted of 1000 excitatory and 800 inhibitory neurons as default (we decreased this number in the simulation of optogenetic inhibition). The membrane potential of each neuron was modeled according to the MP neuron membrane properties (Wang and Sun, 2021) based on the Hodgkin-Huxley model as following:

$$\frac{dV}{dt}=\frac{gl\cdot (El-V)-g_{Na}\cdot m^{3}\cdot h\cdot \left(V-E_{Na}\right)-g_{K}\cdot n^{4}\cdot \left(V-E_{K}\right)+g_{e}\cdot \left(E_{e}-V\right)+g_{i}\cdot \left(E_{i}-V\right)+I_{ex}\cdot t_{s}}{C_{m}},$$

$$\frac{dg_{e}}{dt}=-\frac{g_{e}}{\tau_{e}},$$

$$\frac{dg_{i}}{dt}=-\frac{g_{i}}{\tau_{i}},$$

where the excitatory and inhibitory synaptic time constants ***τ***_***e***_ and ***τ***_***i***_ were set as 5 and 10ms, respectively. Other metrics were adjusted to adapt the inter-spike-intervals of L5 mPFC MP neuron, which proposed to be a functional and dominate interneurons that bridge the gap between deep brain regions and motor cortex (Wang and Sun, 2021). The membrane potential ***V*** was initiated randomly at a range from −65mV to −63mV. The excitatory conductance was set at a range from 0 to 0.06ns and the inhibitory counterpart was in 0 to 1.5ns.

The synapse action was dependent on the usage *u* and availability *x* of released neurotransmitter before and after an action potential as following: $\frac{du}{dt}=-\omega_{f}\cdot u$, $\frac{dx}{dt}=\omega_{d}-\omega_{d}\cdot x$, where the facilitation rate was set as 3.33/s and the depression rate was set as 2/s. The rest synaptic release probability was set as 0.6. The probability of excitatory connection of the network was given as 0.05 and the inhibitory counterpart was 0.2.

To generate lick raster data from the network, we first assumed that one single lick cycle is governed by a rotational neural dynamic (Linden et al., 2022). Then we divided one cycle of the lick into nine phases. The triggering probability *P* of a lick signal was calculated from the network through a decision algorithm:

$$P(t)=\{\begin{array}{l} 1,\prod_{\theta =0}^{2\pi }\left(\sum_{i=1}^{N}\varphi_{\theta i}(t-280)\right)>0\;\&\;S\left(\sum_{i=1}^{N}\varphi_{\theta i}(t-280)\right)<30\\ 0,\prod_{\theta =0}^{2\pi }\left(\sum_{i=1}^{N}\varphi_{\theta i}(t-280)\right)=0\;or\;S\left(\sum_{i=1}^{N}\varphi_{\theta i}(t-280)\right)\geq 30 \end{array},$$

where *S* represents the standard deviation of the spike counts in whole phases.

### Statistical test

To evaluate the statistical significance of the association between the neural coding of valence and movement phases, Chi-square test was used to compare the percentage between overlap and non-overlap group (Fig 1E). For comparison of the mean of facial and locomotor activity (Fig S1K–N and Fig S9E & H), comparison of the mean of licking frequency at different sessions (Fig S1C–F), and comparison of TSPE (Fig S5), we used two-tailed Wilcoxon signed rank test. For evaluation of the slope of spike counts trend line, we used one sample t test (Fig S4E). The rest of statistical tests were two sample t test.

## Supplementary Material

1
